# Prevalence of unfavorable outcome in acute poisoning and associated factors at the University of Gondar comprehensive specialized hospital, Gondar, Northwest Ethiopia: a hospital-based cross-sectional study

**DOI:** 10.3389/fpubh.2023.1160182

**Published:** 2023-06-08

**Authors:** Leta Gemeda Waktola, Endalkachew Belayneh Melese, Nebiyu Mesfin, Kassaye Demeke Altaye, Gebrehiwot Lema Legese

**Affiliations:** Department of Internal Medicine, School of Medicine, College of Medicine and Health Sciences, University of Gondar, Gondar, Ethiopia

**Keywords:** poisoning, unfavorable outcome, Gondar, Ethiopia, associated factors

## Abstract

**Background:**

Acute poisoning is a medical emergency in which the toxic effects occur almost immediately, usually within hours from the time of exposure, and can result from exposure to excessive doses of any chemical. It is the common cause of emergency admission, which may result in morbidity and mortality. There are a lot of factors that are associated with an increased magnitude of mortality and complication. Therefore, this study was carried out to assess the clinical characteristics of patients, unfavorable outcomes of acute poisoning, and associated factors to improve the quality of care, resource utilization, and decrease mortality.

**Objective:**

This study aimed to assess the outcome and associated factors among acute poisoning patients at the University of Gondar Comprehensive Specialized Hospital, Gondar, Northwest Ethiopia (2021).

**Methods:**

A prospective follow-up study was conducted from January 2021 to September 2021 at the University of Gondar Comprehensive Specialized Hospital, Gondar, Northwest Ethiopia. Data were collected using a comprehensively organized and pretested interviewer-administered questionnaire. The data were entered using EPI data version 4.6.0 statistical software and then exported to Stata 14 for analysis. The data were analyzed for descriptive statistics. Statistical analysis was performed using bivariate and multivariate logistic regression models to identify factors associated with the unfavorable outcome of acute poisoning. The result is presented in the form of tables, figures, and text using frequencies and summary statistics such as mean, SD, median, IQR, and percentage.

**Result:**

A total of 233 patients were included in the study. The prevalence of unfavorable poisoning outcomes in acute poisoning was 17.6% (95% CI: 13.2, 23.1). In the multivariate logistic regression analysis, known chronic medical comorbidities [AOR: 3.846 (1.619, 9.574); value of *p*: 0.014] and hospital stay of less than 48 h [AOR: 6.57 (2.03, 21.273); value of *p*: 0.002] were found to be independent factors associated with unfavorable outcomes in acute poisoning.

**Conclusion:**

The magnitude of unfavorable poisoning outcomes was high in patients with acute poisoning. Having known medical comorbid illness and short hospital stay of less than 48 h were found to be associated with unfavorable outcomes.

## Introduction

Acute poisoning is one of the commonest causes of emergency admission which is exposure to poison for a short period (less than 24 h) through any route, intentionally or unintentionally. In East Africa, the burden of acute poisoning is increasing due to changes in lifestyle, availability of chemicals, and social behavior (1), which similarly works for Ethiopia (2).

Though the fatality rate of acute poisoning varies owing to differences in socioeconomic, cultural, and healthcare facility levels of the country, worldwide it is responsible for more than 1 million morbidities, with a 20% fatality rate (3). Acute poisoning-related morbidity and mortality are much worse in low- and middle-income countries (LMICs) because of insufficient drug and chemical regulations, lack of surveillance systems, easy access to more toxic drugs or chemicals, and poor healthcare services (4).

While the common cause of poisoning-related emergency admission in developed nations is therapeutic drug poisoning (1, 5, 6), organophosphate, and household cleansing, alkaline phosphide takes the majority of the cause in developing countries (7, 8). Similarly, a study conducted in Ethiopia showed that the commonest causes of emergency visits due to acute poisoning were household cleansing agents, organophosphates, rodenticides, and drugs (9–13). Though there is inconsistency, the determinants of unfavorable treatment outcomes following acute poisoning are a long hospital stay, being female, intentional poisoning, organophosphate poisoning, and timely arrival to the hospital (12, 14, 15).

Unlike developed nations, in which the health impact of acute poisoning is well documented (16), the risks and impacts posed by acute poisoning are not predictable in developing countries, which is bound to determine evolving deathtraps, focus on prevention and education programs, and guide clinical research (17, 18).

Though there are a few studies on acute poisoning in Ethiopia, there are scarce statistics on acute poisoning in the study area, the sample size of the previous study was small, and almost all are retrospective studies that are weak to show the determinants of unfavorable treatment outcomes. Additionally, previous studies were inconsistent, and the determinant factors of unfavorable treatment outcomes of acute poisoning were different across the country. Hence, this prospective cross-sectional study is aimed to assess the treatment outcome of acute poisoning and identify factors associated with unfavorable treatment outcomes among patients who had been admitted to the emergency study area.

## Methods and participants

### Study area, period, and study design

A cross-sectional study was conducted from 1 January to 30 September 2021 at University of Gondar Comprehensive Specialized Hospital (UoGCSH) emergency department. The hospital is located in Gondar city, central Gondar zone, northwestern Ethiopia, which is 663 km Northwest of Addis Ababa. UoGCSH is the oldest hospital in Gondar town and is the only referral hospital for more than 13 million people from the five zones of Northwest Ethiopia, with a capacity of 950 beds. The emergency department is one of the clinical service areas of the hospital and is divided into WHO color-coded treatment zone of red (10 beds), orange (20 beds), yellow (15 beds), and green (10 beds) as well as additional areas of decontamination isolation and procedure room. It is staffed with three emergency and critical care medicine specialists, eight residents, 20 medical interns, and 34 nurses. The emergency department receives an average of 80 patients per day and 28,000 per year. When patients arrived at triage, they will be categorized based on the cape triage protocol. It has also a well-equipped ICU with a portable dialysis machine, portable ultrasound, and X-ray.

### Source and study population

The source population of this study was all the patients who visited the emergency department of the University of Gondar Comprehensive Specialized Hospital, and the study population was all the patients who visited the emergency department due to acute poisoning during the data collection period.

### Sampling size and sampling procedure

Sample size is calculated using single population proportion formula with assumption of a proportion of mortality of 18.6% from a study done at Debre Tabor, Ethiopia (13), 95%CI, margin of error=5%, zα/2= 1.96, *n* = (Z ά/2)2, and p (1-p)/d2 where p is 0.186, 95% CI, margin of error = 5%, zα/2 = 1.96.

The total sample size was 233, and then, for a 10% non-response rate, the sample size was 254. Since the study population is finite, all patients who visited the emergency department with acute poisoning within the study period were included. There were no non-respondents.

### Data collection

Data were collected using a structured questionnaire by one medical doctor and one nurse after orientation on how to fill out the questionnaire. The questionnaires include sociodemographic factors (age, sex, residence, marital status, and occupation), baseline information (with whom the patient is living, presence of comorbid illness, known history of psychiatric illness, and previous history of suicide attempt), the intention of poisoning, types of poisoning, the root of exposure, time lapse, vital signs at presentation, laboratory results at admission, treatment the patient receives during the course of hospitalization, total duration of hospital stay, outcome, and the immediate cause of death.

### Data quality assurance

The structured questionnaire was developed from different related studies. Data quality was assured by checking the completeness of filled questionnaires and supervision. The questionnaire was pretested on 10% of acute poisoning patients of the total sample size. Those patients who were included in the pretesting were not included in the actual data collection. After the collection of data, a specific marker “poisoning” on the chart was used to avoid repetition.

### Data processing and analysis

The data were entered and cleaned with Epidata version 4.6.0.2 and analyzed by Stata version 14 software packages. The data were analyzed for descriptive statistics and bi-variable logistic regression to determine the effects of various factors on the outcome variable. After that, a multivariable logistic regression analysis was performed to control the confounding effect on those variables with a value of *p* of <0.2. Variables with a value of *p* of less than 0.05 were taken as statistically significant factors for unfavorable outcomes. OR with 95% CI was used to show the degree of association between the independent and dependent variables.

### Operational definition

#### Acute poisoning

Acute poisoning is exposure to poison for a short period (less than 24 h) through any route, intentionally or unintentionally.

#### Outcome

In this study, the outcome was interpreted as a favorable outcome if they were discharged or improved or as an unfavorable outcome if they went against medical advice or died in the hospital.

#### Time lapse

The time between poison exposure and the emergency visit.

### Ethics consideration

**Figure 1:**
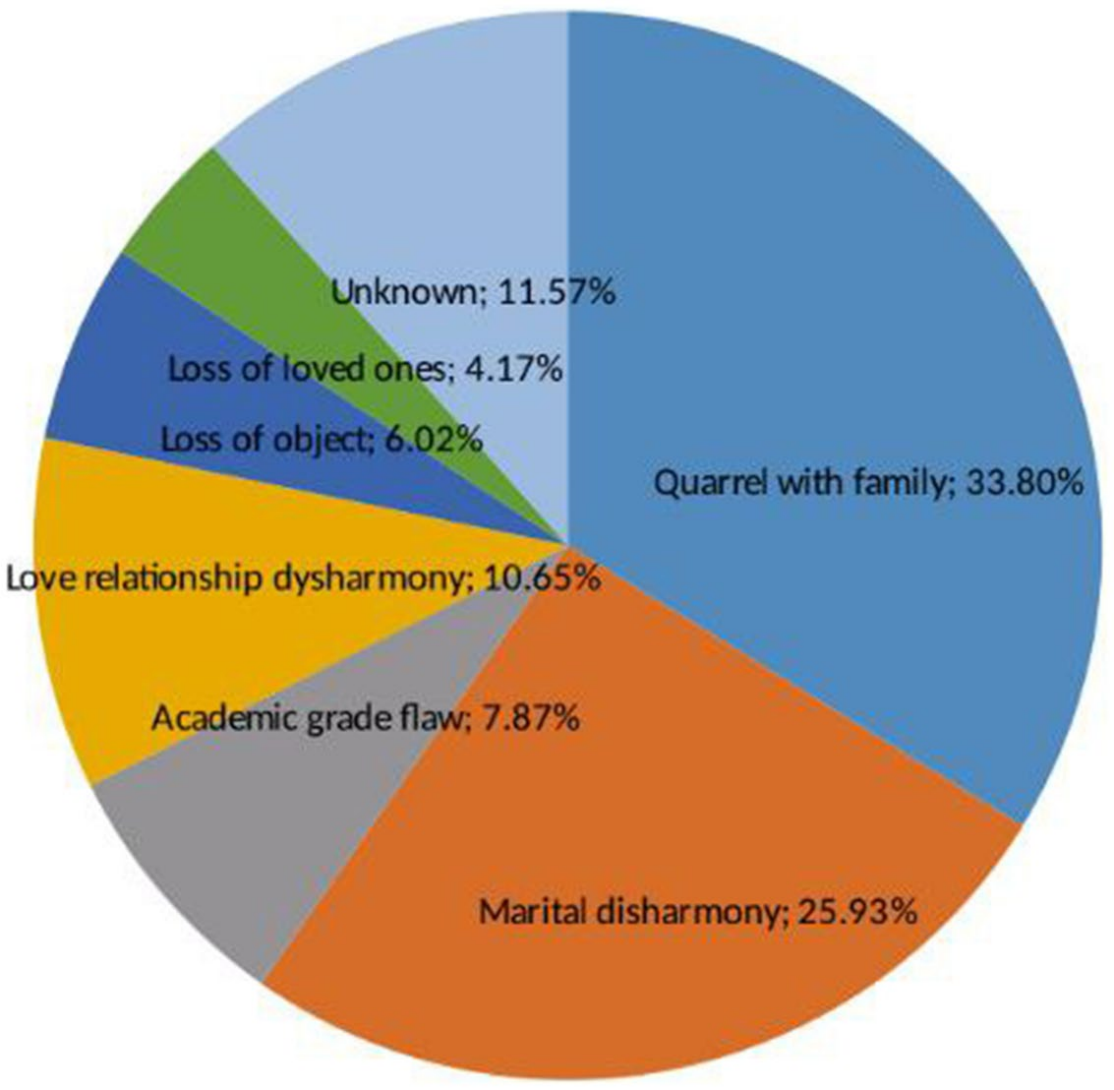
Reasons of acute poisoning at UOG comprehensive specialized hospital, 2021 (*n* = 233).

The study was conducted after ethics approval was obtained from the University of Gondar, College of Medicine, and Health Sciences Institutional Research Ethics Review Committee, with the reference number SoM Ref.No./614/05/2021. Before data collection, verbal informed consent was obtained from all the participants. A record chart number was used as a patient identifier, and the name of the patients was excluded to keep and respect the confidentiality of all information obtained. Patients who were not willing to participate in the study were not denied the routine care they get at the hospital (Figure 1).

## Results

### Sociodemographic characteristics

A total of 233 acute poisoning cases visited the emergency department during the study period. A higher proportion of 148 (63.52%) of the acutely poisoned patients were women. The median age of patients with acute poisoning was 23 years (IQR: 18, 60), of which young adults (18–25 years of age) made up the highest proportion (55.36%). More than half [*n* = 128 (54.94%)] of the patients live in urban areas as described in Table 1.

**Table 1:** Socio-demographic characteristics of patients with acute poisoning at UOG Comprehensive Specialized Hospital, 2021 (*n* = 233).

Variable	Categories	Freq.	Percent
Age (years)	18–25	129	55.36
	26–35	67	28.76
	36–45	20	8.58
	≥46	17	7.29
Sex	Male	85	36.48
	Female	148	63.52
Residency	Urban	128	54.94
	Rural	105	45.06
Marital status	Married	114	48.93
	Single	110	47.21
	Divorced and widowed	9	3.87
Educational status	Unable to read and write	65	27.90
	Able to read and write but no formal education	16	6.87
	Elementary school	64	27.47
	High school and preparatory	67	28.76
	College and above	21	9.01

### Base line information

Regarding the living condition of the patients, 122 (48.06%) of them reported that they were living with their families. Only eight (3.43%) patients had comorbidities. Most of the patients [*n* = 214 (96.85%)] had no previous history of psychiatric illness. In this study, 4.29% of the poisoning cases had a history of previous suicidal attempts, among which 55.56% had received formal counseling (Table 2).

**Table 2:** Base line information of the acute poisoning patients at UOG Comprehensive Specialized Hospital, 2021 (*n* = 233).

Variables	Categories	Freq.	Percent
Whom is the patient living with	Alone	21	9.01
	With family/friends	212	90.99
Presence of comorbid illness	Yes	8	3.43
	No	225	96.57
Known history of psychiatric illness	Yes	19	8.15
	No	214	91.85
Previous history of suicide attempt	Yes	10	4.29
	No	223	95.71

### Intention and route of acute poisoning

In this study, 216 (92.70%) patients were acutely poisoned intentionally (deliberate self-harm), while 15 (7.3%) of the cases accounted for unintentional, of which 5.58% was accidental acute poisoning. The oral route is the most common route of exposure 223 (95.71%). The most common reasons for acute poisoning were quarrels with family (33.8%) and marital disharmonies (25.93%), as described in Figure 1.

### Common poisoning agents

**Figure 2:**
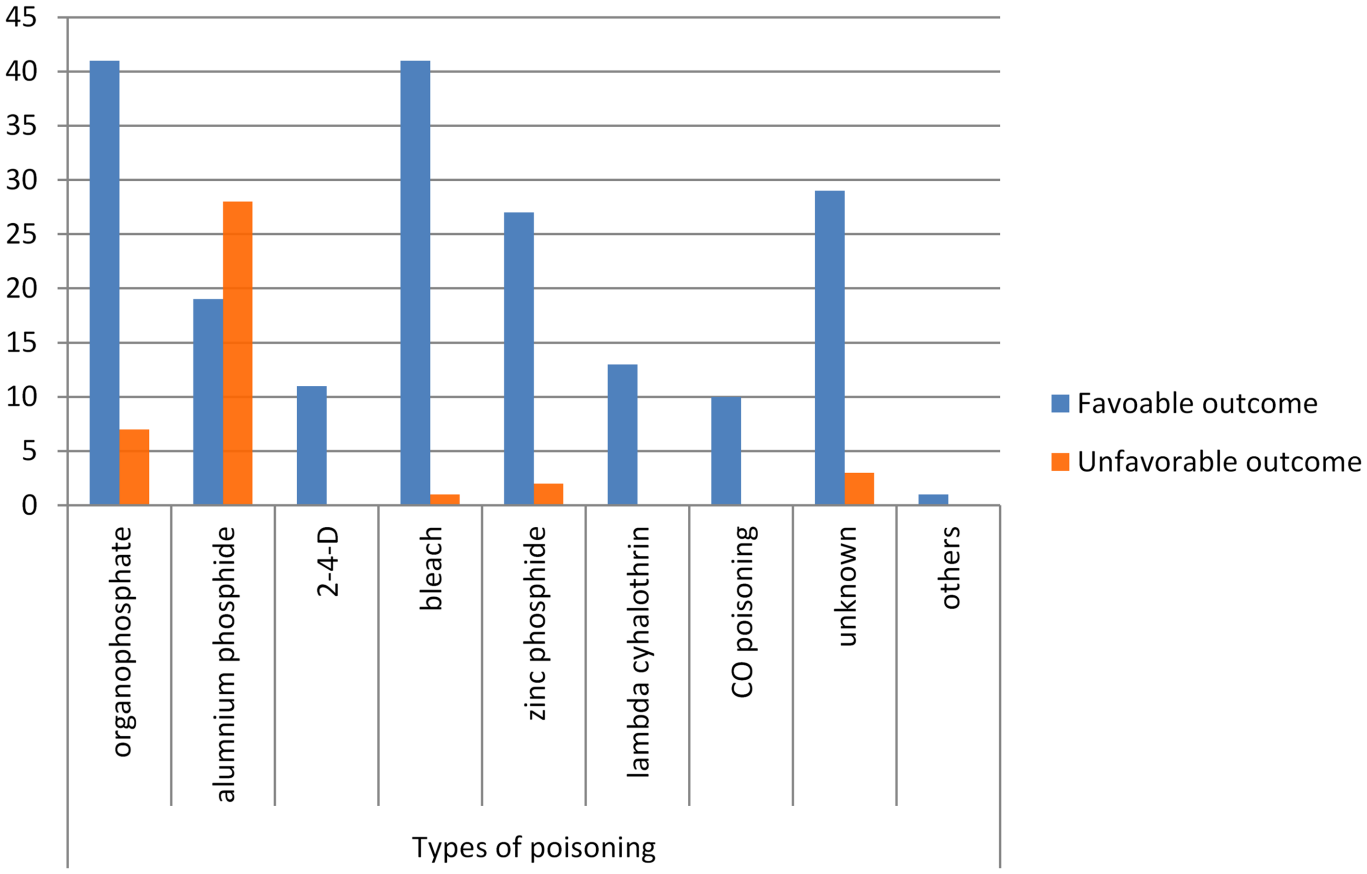
Category of common poisoning agents identified at UOG comprehensive specialized hospital, 2021 (*n* = 233).

Of the total acute poisoning cases, the most common categories of poisoning agents implicated were organophosphate 48 (20.69%), aluminum phosphide 47 (20.26%), and bleach 42 (18.10%) (Figure 2).

### Patients’ clinical characteristics

Most of the patients had delayed presentation with >2 h time lapse 158 (67.81%). Mean blood pressure at presentation was 104/65 mmHg, where 29 (12.45%) patients had BP of less than 90/60 at presentation. Among the total number of patients, 28 (12.01%) had known comorbidities, the commonest being a history of psychiatric illness 19 (8.15%) (Table 3).

**Table 3:** Vital signs at presentation of acute poisoning patients at UOG Comprehensive Specialized Hospital, 2021 (*n* = 233).

Variable	Categories	Freq.	Percent
SBP (mmHg)	<90	29	12.45
	90–140	196	84.12
	≥140	8	3.43
DBP (mmHg)	<60	32	13.73
	60–90	194	83.26
	>90	7	3.00
Pulse rate (bpm)	<60	3	1.29
	60–100	129	55.36
	>100	101	43.35
SPO2 (%)	<90	32	13.73
	> = 90	201	86.27

### The intervention was conducted at the admission

A specific antidote was given for 43 (18.45%) patients, of which atropine [*n* = 36 (15.45%)] is the most common antidote given. Most of the patients received IV fluid [148 (63.52)], and 32 (13.73%) patients received inotropes for hemodynamic instability. The majority of the patients were given antacids (77.68%), followed by antiemetics (18%) (Table 4).

**Table 4:** Treatment given during course of hospitalization for acute poisoning patients at UOG comprehensive specialized hospital, 2021 (*n* = 233).

Variables	Categories	Frequency	Percent
Antidote	Yes	43	18.45
	No	190	81.55
INO2	Yes	14	6.01
	No	219	93.99
IV fluid	Yes	148	63.52
	No	85	36.48
Inotropes	Yes	32	13.73
	No	201	86.27
Antibiotics	Yes	14	6.01
	No	219	93.99
Hemodialysis	Yes	1	0.43
	No	232	99.57
Mechanical ventilation	Yes	1	0.43
	No	232	99.57
Antacids	Yes	181	77.68
	No	52	22.32
Antiemetic (metoclopramide)	Yes	43	18.45
	No	190	81.55

### Outcomes of acute poisoning

**Figure 3:**
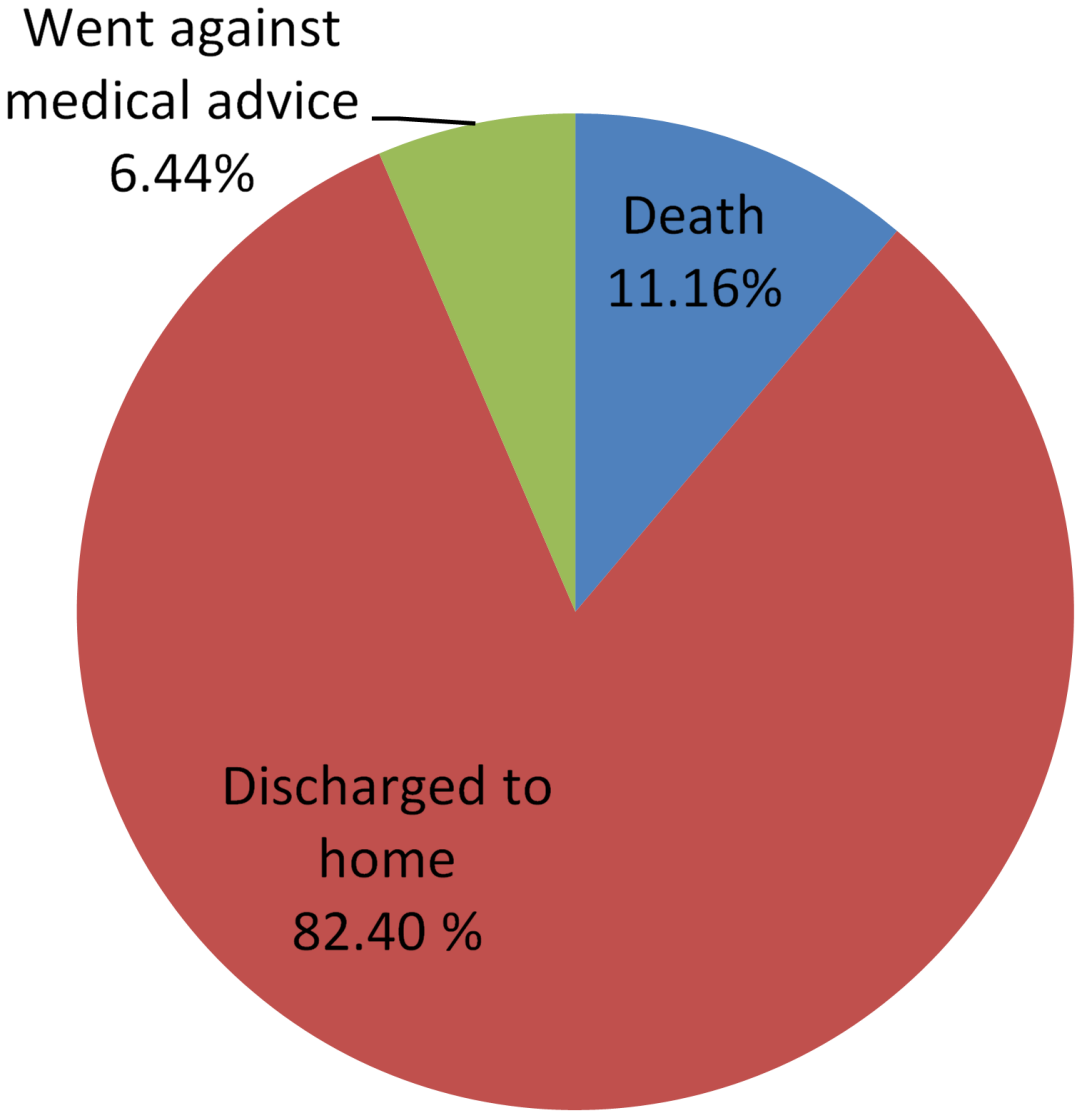
Outcome of acute poisoning at UOG comprehensive specialized hospital, 2021 (*n* = 233).

Of the 233 poisoning cases, 192 (82.40%) patients survived and were discharged to home, and the remaining 41 [17.6% (95% CI: 0.132, 0.231)] patients had unfavorable outcomes, of which the case fatality rate accounts for 11.16% (*n* = 26), as shown in Figure 3. Of the 26 deaths reported, aluminum phosphide represents the highest percentage 21 (80.76%), followed by organophosphate 3 (11.54%) and zinc phosphide 2 (7.69).

In binary logistic regression, age, sex, residency, educational status, presence of comorbidities, time lapse, IV fluid, and total duration of hospital stay were statistically significant at a value of *p* of 0.2 and less than that.

The result of the multivariate logistic regression analysis showed that the independent factors associated with unfavorable outcomes were having known chronic medical comorbidities [AOR: 3.846 (1.619, 9.574); value of *p*: 0.014] and hospital stay of <48 h [AOR: 6.570 (2.029, 21.273); value of *p*: 0.002] (Table 5).

**Table 5:** multivariate logistic regression analysis of factors associated with unfavorable outcome of acute poisoning at UOG Comprehensive Specialized Hospital, 2021 (*n* = 233).

Variables	Categories	Outcome		COR [95%]	AOR [95%]	*p*-value
		Unfavorable	Favorable			
Age (years)	18–25	20	109	1	1	
	26–35	11	56	1.070 (0.479, 2.390)	0.802 (0.301, 2.145)	0.659
	36–45	5	15	1.816 (0.593, 5.561)	1.28 (0.324, 5.124)	0.727
	45+	5	12	2.270 (0.721, 7.150)	1.039 (0.243, 4.446)	0.958
Sex	Male	17	68	1	1	
	Female	24	124	0.774 (0.389, 1.540)	0.828 (0.376, 1.82)	0.638
Residency	Urban	15	113	1	1	
	Rural	26	79	2.479 (1.234, 4.980)	2.204 (0.864, 5.651)	0.1
Educational status	Unable to read and write	16	49	1	1	
	Able to read and write no formal schooling	5	11	1.392 (0.419, 4.614)	2.077 (0.547, 7.889)	0.283
	Elementary School	10	54	0.567 (0.235, 1.366)	0.663 (0.208, 2.116)	0.487
	High School and primary	8	59	0.415 (0.163, 1.051)	0.54 (0.154, 1.896)	0.336
	College and above	2	19	0.322 (0.067, 1.537)	0.386 (0.057, 2.625)	0.33
Presence of comorbidities	No	35	172	1	1	
	Yes	6	20	2.219 (1.338, 4.737)	3.846 (1.619, 9.574)	0.014*
Time lapse	≤ 2 h	9	66	1	1	
	>2 h	32	126	1.862 (0.839, 4.133)	1.528 (0.61, 3.826)	0.365
IV fluid	Yes	31	117	1	1	
	No	10	75	0.503 (0.233, 1.086)	0.642 (0.276, 1.493)	0.303
Total duration	≥48 h	5	65	1	1	
of stay	<48 h	36	127	3.685 (1.380, 9.838)	6.570 (2.029, 21.273)	0.002**

*-significant results, 1-reference category **p-value < 0.001, *p-value ≤ 0.05.

## Discussion

In this study, most patients with acute poisoning were young adults (18–25 years). This result was comparable with the studies conducted in Ethiopia (19), Kenya (20), China (21), and Iran (22). The reason for the high rate of poisoning in young adults may be explained by their vulnerability to stressful life situations such as unemployment, marital problems, failure or frustration in love or job or examinations, inability to fulfill the parents’ expectations, and immaturity to cope up with those situations; thus, they would be easily tempted to attempt suicide.

The gender distribution of acute poisoning in the current study revealed female predominance. This finding appears to be concordant with other studies conducted in JUSH, Ethiopia (52.4%) (23), and Tehran, Iran (55.7%) (22). The higher proportion of women being acutely poisoned might be because some situations such as family disharmony could be frustrating for them and they might attempt suicide. However, other studies have indicated a higher incidence of acute poisoning in men, in Zambia (52%) (24), Kenya (58.33%) (25), and Iran (26). This may be related to more exposure of males to occupational hazards and stress.

In this study, the intentional manner of poisoning type was the most common 216 (92.70%). Similarly, other studies have confirmed the situation in different parts of Ethiopia and China (21, 27–29). This could be explained by poisoning, which is a common method of attempting suicide because many people believe that poison can terminate life with minimal suffering.

In this study, the most commonly consumed for acute poisoning was organophosphates 48 (20.69%), followed by aluminum phosphide 47 (20.26%), which is similar to other studies in Dessie–Ethiopia (30), Kenya (20), and India (31). In contrast, in studies conducted in Addis Ababa, Ethiopia (15), and Botswana (32), household chemicals were the commonest agents used for poisoning, and in Taiwan (33) and Turkey (6), drugs were the commonest. Probably, some of the reasons why organophosphates are commonly employed for self-poisoning in this study are increased access to pesticides due to the rampant use of pesticides in our country’s agricultural industry, easy availability, and lack of adequate regulations regarding their purchase and use.

The oral route is the most common route of exposure to poisoning agents encountered in this study 223 (95.71%). This was similar to other studies conducted in Nekemte, Ethiopia (34), Turkey (35), and Tanzania (36). This is explained by a specific poisoning agent and available formulation related to the agent. Inhalation accounts for 10 (4.29%) cases of carbon monoxide poisoning in this study. It was also reported as 2.1% in the study by Satar et al. (5) and 8.7% in the study by Sacak et al. Turkey (37). This occurred when there is incomplete combustion of organic material leading to the formation of carbon monoxide. The most common sources of CO poisoning are faulty or inadequately ventilated houses with the combustion of charcoal as an energy source of heat.

In this study, 17.6% (95% CI: 0.132, 0.231) of the participants had unfavorable outcomes, which is comparable to the studies conducted in Debretabor, Ethiopia 18.6% (38), India 13.6% (39), Iran 19.5% (40), and Sri Lanka 21.5% (41). On the opposite of this study, a study conducted in central Ethiopia (15) was 8.6%, and a study conducted in Botswana (32) was found to be 2.6%. This might be due to household type of poisoning, early and easy access to health facilities, and a relatively good setup in the health facilities.

The reason for the higher mortality in this study because most of the cases are aluminum phosphide poisoning which is more fatal even with a small dose and having no the antidote may be the other reason, as 68.29 % of the patients who took aluminum phosphide had unfavorable outcomes, which was also seen in other study in India (42).

## Strengths and limitations of the study

### The strength of the study

The prospective nature of the study design enabled the researchers to deal with the actual spectrum and nature of poisoning prevalence and its outcomes which might otherwise be impossible to achieve using the retrospective type.

### Limitations of the study

As the findings are obtained from one hospital, the results may not be the true reflection of the problem of the general population. In addition, as patients were followed up only during their stay at the hospital, the study did not reflect the fate of patients who were discharged against medical advice.

## Conclusion

The rate of unfavorable poisoning outcomes was high in patients with acute poisoning. Having known medical comorbid illness and hospital stay of <48 h were found to be associated with unfavorable outcomes.

## Data availability statement

The original contributions presented in the study are included in the article/supplementary material, further inquiries can be directed to the corresponding author.

## Ethics statement

Ethical approval was obtained from the University of Gondar, College of Medicine and Health Sciences, School of Medicine, Ethical Review committee with the reference number SoM Ref.No./614/05/2021. Before data collection, verbal informed consent was obtained from all participants to participate in this study. Record chart number was used as a patient identifier and the names of the patients were excluded to keep and respect the confidentiality of all information obtained.

## Author contributions

LGW, KDA, NM, EBM, and GLL are involved in conceiving the idea, study design, data analysis, and interpretation, and managing the overall progress of the study and manuscript preparation. LGW, KDA, and NM are equally involved in the study design, and follow-up of the study. Both EBM and GLL equally contributed to data analysis and interpretation, and in revising the manuscript. The final manuscript was read and approved by all the authors.

## Conflict of interest

The authors declare that the research was conducted in the absence of any commercial or financial relationships that could be construed as a potential conflict of interest.

## Publisher’s note

All claims expressed in this article are solely those of the authors and do not necessarily represent those of their affiliated organizations, or those of the publisher, the editors and the reviewers. Any product that may be evaluated in this article, or claim that may be made by its manufacturer, is not guaranteed or endorsed by the publisher.
